# Necrotizing Pneumonia in an Elderly Patient With Chronic Obstructive Pulmonary Disease (COPD): A Case Report

**DOI:** 10.7759/cureus.102563

**Published:** 2026-01-29

**Authors:** Andreia Salgadinho Machado, Raquel Borrego, Marta Roldão, Marta Anastácio, Sandra André

**Affiliations:** 1 Internal Medicine, Unidade Local de Saúde de Lisboa Ocidental, Lisbon, PRT; 2 Internal Medicine, Centro Hospitalar Lisboa Ocidental, Lisbon, PRT; 3 Pulmonology, Unidade Local de Saúde de Lisboa Ocidental, Lisbon, PRT; 4 Pulmonology, Unidade Local de Saude de Lisboa Ocidental, Lisbon, PRT

**Keywords:** community-acquired pneumonia, copd: chronic obstructive pulmonary disease, multidrug-resistant bacteria, necrotizing pneumonia, staphylococcus aureus

## Abstract

Necrotizing pneumonia (NP) is a severe complication of bacterial pneumonia, characterized by progressive lung necrosis and cavitation. Although advances in imaging and supportive care have improved outcomes, NP remains difficult to manage due to poor antibiotic penetration, frequent polymicrobial infection, and the lack of standardized treatment protocols.

An 85-year-old man with chronic obstructive pulmonary disease (COPD) (Global Initiative for Obstructive Lung Disease (GOLD) group C) and a 40-pack-year former smoking history, managed with long-acting beta-agonist/long-acting muscarinic antagonist (LABA/LAMA), was admitted with acute dyspnea and productive cough. Imaging showed left-sided pneumonia, and methicillin-sensitive *Staphylococcus aureus *(MSSA) was isolated. Amoxicillin-clavulanate led to transient improvement, but recurrent fever, worsening hypoxemia, and new cavitary lesions developed. Antimicrobial therapy was broadened to piperacillin-tazobactam, vancomycin, and voriconazole, without clinical stabilization. After empiric meropenem, the patient improved, completed 21 days of treatment, and fully recovered. However, without microbiologic confirmation, a direct causal link to meropenem cannot be established.

This case highlights the diagnostic and therapeutic challenges of necrotizing pneumonia in elderly COPD patients, particularly when differentiation between progression, relapse, or superinfection is hampered by inconclusive microbiological findings. Although clinical improvement followed escalation to meropenem, the evidence does not permit attribution of recovery solely to this intervention. These findings reinforce the need for frequent reassessment, individualized treatment strategies, and caution in generalizing therapeutic recommendations from individual case reports.

Early identification of necrotizing complications and timely modification of antimicrobial therapy are critical. Broad-spectrum antibiotics, including carbapenems, should be reserved for cases with compelling clinical or microbiologic evidence of multidrug resistance, rather than being used routinely based on isolated case experiences.

## Introduction

Necrotizing pneumonia (NP) is a severe complication of bacterial pneumonia, resulting in pulmonary tissue necrosis and cavity formation. Management of NP is challenging due to the involvement of the most frequently implicated pathogens, *Streptococcus pneumoniae, Staphylococcus aureus*, and *Klebsiella pneumoniae*, limited antibiotic penetration to affected areas, and the frequent failure of laboratory tests to identify the causative organism [1]. 

The clinical presentation of NP ranges from mild symptoms to life-threatening infection, depending on the extent of pulmonary involvement and the patient’s comorbidities. Older adults and individuals with chronic illnesses are particularly susceptible to severe complications. Prompt recognition and timely escalation of antibiotic therapy are essential to mitigate adverse outcomes [2].

This report presents a case of necrotizing pneumonia in an elderly man with chronic obstructive pulmonary disease (COPD). We highlight the diagnostic and therapeutic challenges encountered and discuss key clinical lessons derived from this case.

## Case presentation

An 85-year-old male with chronic obstructive pulmonary disease (COPD) (Global Initiative for Obstructive Lung Disease (GOLD) group C) and a 40-pack-year smoking history, managed with long-acting beta-agonist/long-acting muscarinic antagonist (LABA/LAMA) therapy, presented to the emergency department on the first day of illness. He reported two days of progressive dyspnea, orthopnea, and productive cough with brownish sputum. Recent, unquantified weight loss was noted, while fever, night sweats, and chest pain were denied. On admission, the patient was tachypneic at rest (24 breaths/min) with an oxygen saturation of 95% on a 2 L/min nasal cannula. He appeared fatigued and dyspneic. Lung auscultation revealed diminished breath sounds, wheezes, and rhonchi over the left upper field, with bilateral crackles. Blood pressure was 144/98 mmHg, heart rate 103 bpm, regular, with a grade II/VI systolic murmur. Bilateral pitting edema was present up to the knees. Laboratory tests demonstrated normocytic normochromic anemia, neutrophilic leukocytosis, elevated C-reactive protein and fibrinogen, increased D-dimers, and mild renal dysfunction. Arterial blood gases on 2 L/min oxygen indicated hypoxemia without hypocapnia or hyperlactatemia (Table 1).

**Table 1:** Baseline laboratory values and arterial blood gas analysis at presentation Abbreviations: pO_2_, partial pressure of oxygen; pCO_2_, partial pressure of carbon dioxide; HCO_3_, bicarbonate.

Laboratory parameter	Normal range	Result
Hemoglobin (g/dL)	12-15.3	10.7
White Blood Cells (x10^9/L)	4.0-11.0	24900
Neutrophils (x10^9/L)	1.9-7.5	21340
Lymphocytes (x10^9/L)	1.0-4.8	1370
Eosinophils (x10^9/L)	0-0.5	500
Platelets (x10^9/L)	150-450	378
C-reactive protein (mg/dL)	<0.5	28.4
Fibrinogen (g/L)	2-4	9.44
D-dimer (ng/mL)	<500	2058
Urea (mg/dL)	16-49	65
Creatinine (mg/dL)	0.5-0.9	1.04
Arterial blood gas		
pH	7.35-7.35	7.37
pO₂ (mmHg)	75–100	74
pCO_2_ (mmHg)	35-45	35
HCO_3_ (mmol/L)	22-26	21
Lactate (mmol/L)	0.5–2.0	1.5

**Figure 1:**
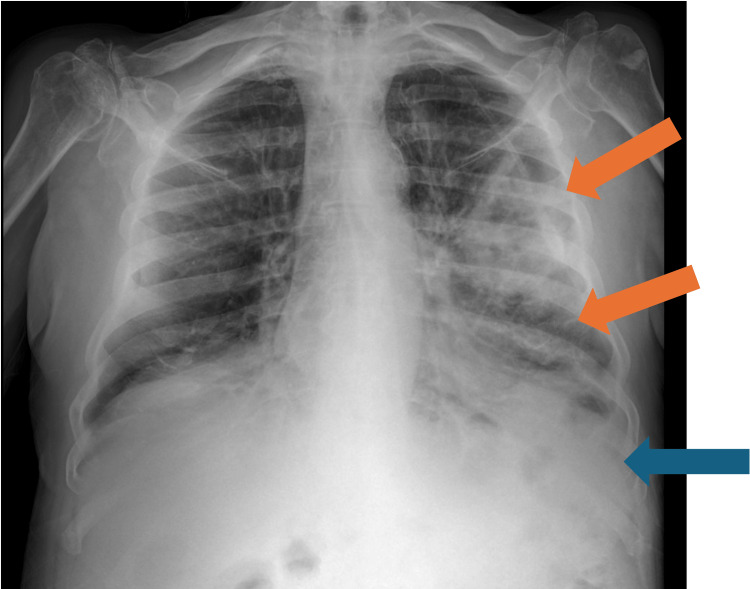
Chest X-ray at admission showing left-sided pneumonia (orange arrows) and pleural effusion (blue arrow)

Chest X-ray demonstrated left-sided pneumonia and a pleural effusion (Figure 1). Nasopharyngeal swabs for SARS-CoV-2 and methicillin-resistant *Staphylococcus aureus* (MRSA), as well as urinary antigen tests for *Streptococcus pneumoniae* and *Legionella pneumophila*, were negative. Microbiological evaluation included the collection of sputum and blood cultures at admission, prior to initiation of antibiotic therapy. Due to the minimal volume of the effusion, thoracentesis for pleural fluid sampling was not performed.

A diagnosis of community-acquired pneumonia with type I respiratory failure was established. Empiric antibiotic therapy with amoxicillin-clavulanate and clarithromycin was initiated. Systemic corticosteroids were administered due to underlying COPD. The CURB-65 score was 5, warranting admission for close monitoring.

Initial clinical and laboratory improvement was observed, including resolution of fever and reduction in inflammatory markers. Sputum culture identified methicillin-sensitive *Staphylococcus aureus* (MSSA), prompting discontinuation of clarithromycin. Blood cultures remained negative.

**Figure 2:**
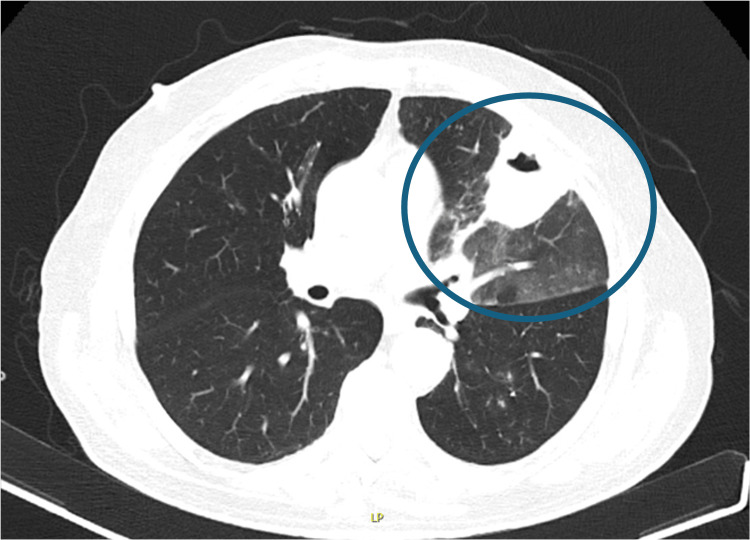
Axial chest CT demonstrating left upper-lobe consolidation with a well-defined cavitary lesion and surrounding parenchymal inflammation (blue circle)

**Figure 3:**
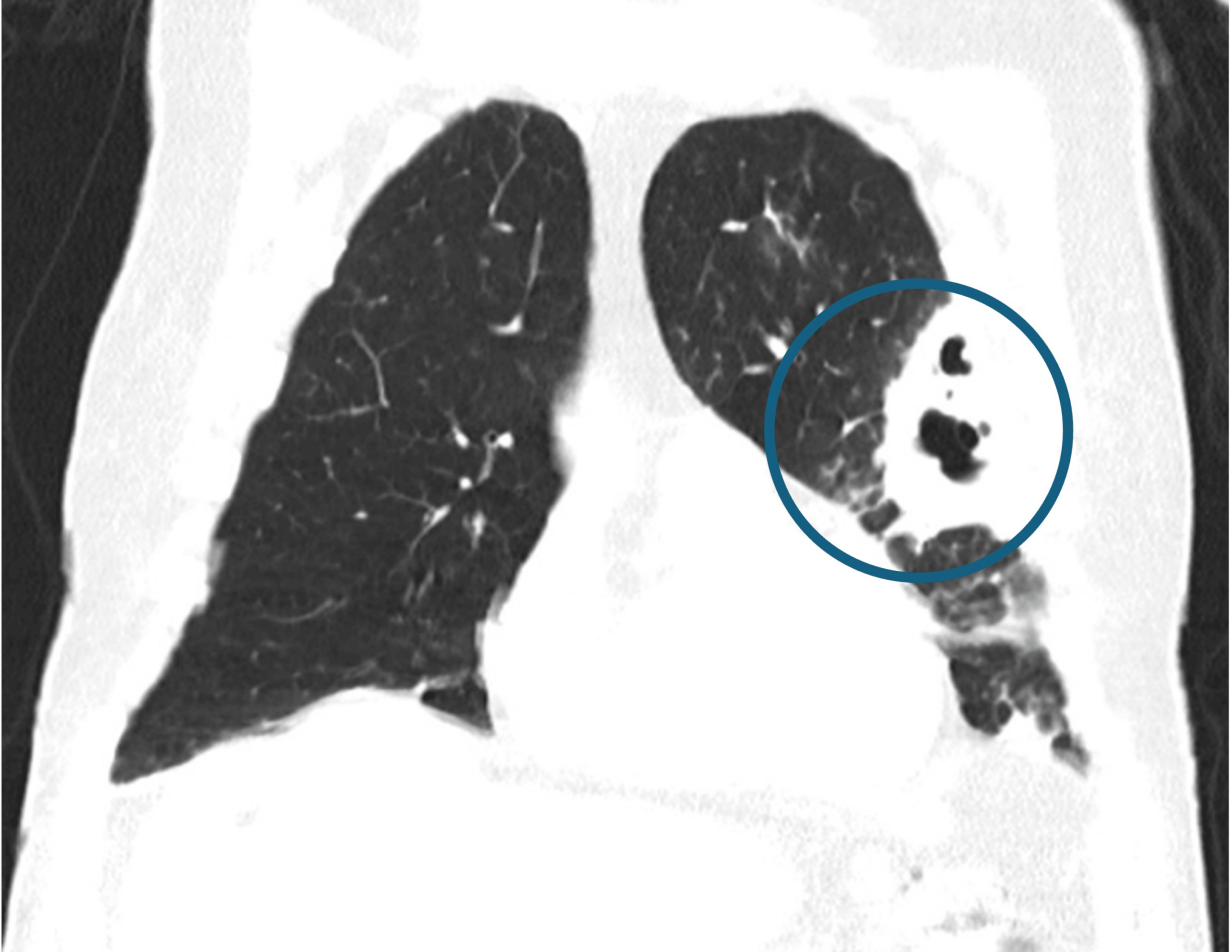
Coronal chest CT demonstrating extensive left upper-lobe consolidation with a well-defined cavitary lesion and surrounding parenchymal inflammation (blue circle)

On day 4 of hospitalization, increasing inflammatory markers prompted bronchoscopy. Three sputum samples were tested for acid-fast bacilli and *Mycobacterium tuberculosis* PCR, all of which were negative. CT imaging revealed peribronchovascular interstitial thickening, ground-glass opacities, left upper lobe consolidation, a juxtaplural cavitary lesion, bilateral “tree-in-bud” micronodules, basal bronchiectasis, and mediastinal lymphadenopathy (Figures 2, 3). Antibiotic therapy was escalated to piperacillin-tazobactam.

**Figure 4:**
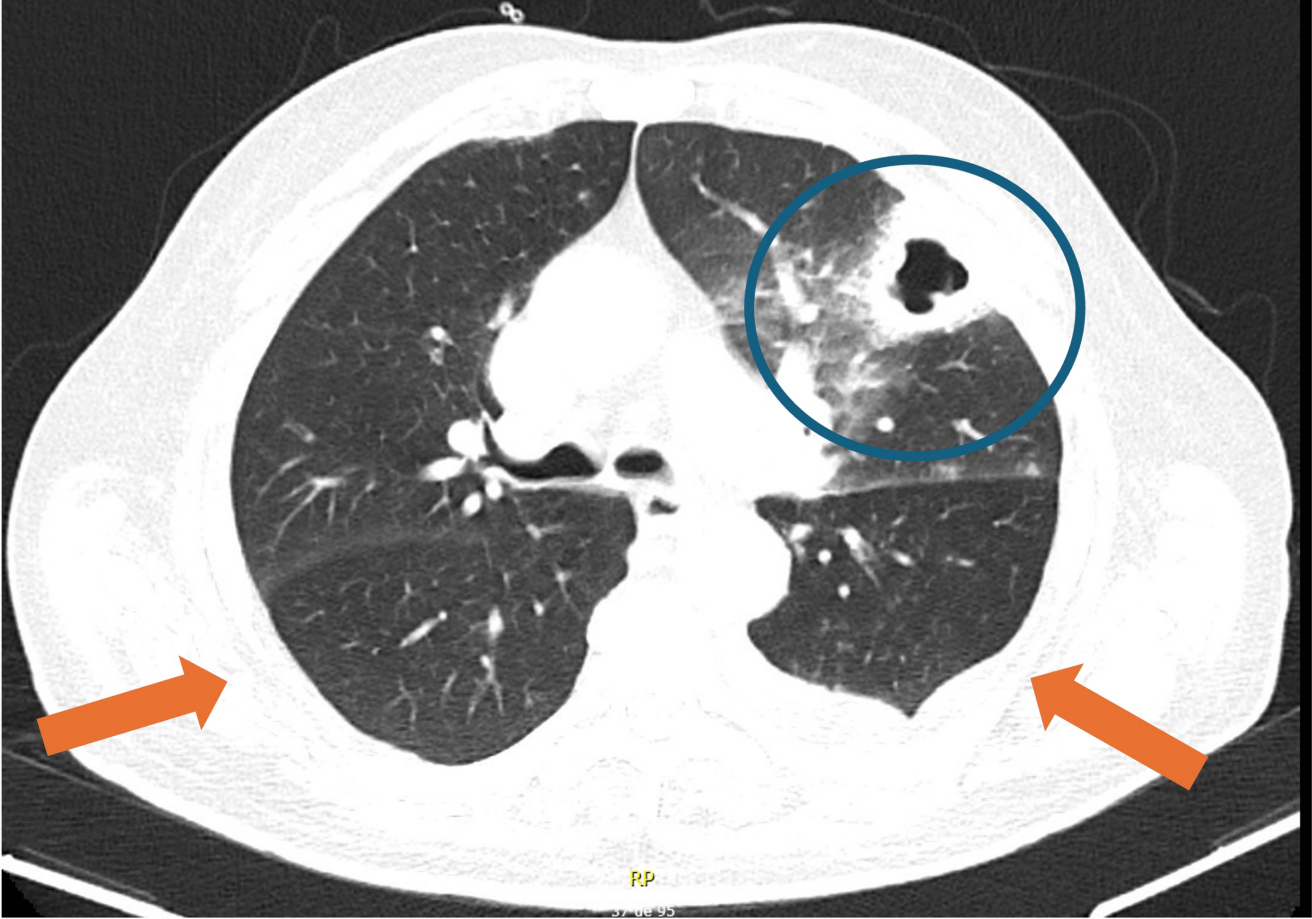
Axial Chest CT showing bilateral progression with cavitary transformation (blue circle) and pleural effusion (orange arrows)

**Figure 5:**
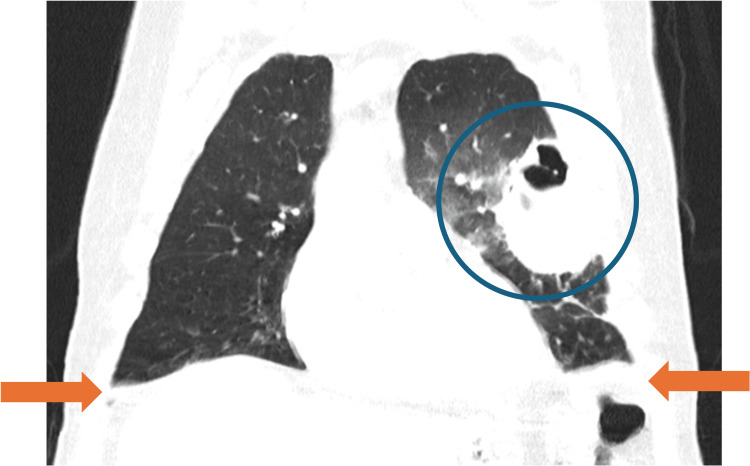
Coronal chest CT demonstrating extensive left upper-lobe consolidation with a large cavitary lesion (blue circle) and pleural effusion (orange arrows)

By day 10, worsening hypoxemia necessitated 40% oxygen via Venturi mask. CT angiography excluded pulmonary embolism and revealed new right-sided consolidations and increased left pleural effusion (Figures 4, 5). Due to radiologic progression, piperacillin-tazobactam was discontinued, and therapy was escalated to vancomycin and voriconazole. The patient was transferred to the Non-Invasive Ventilation Unit (NIVU) on day 14.

**Figure 6:**
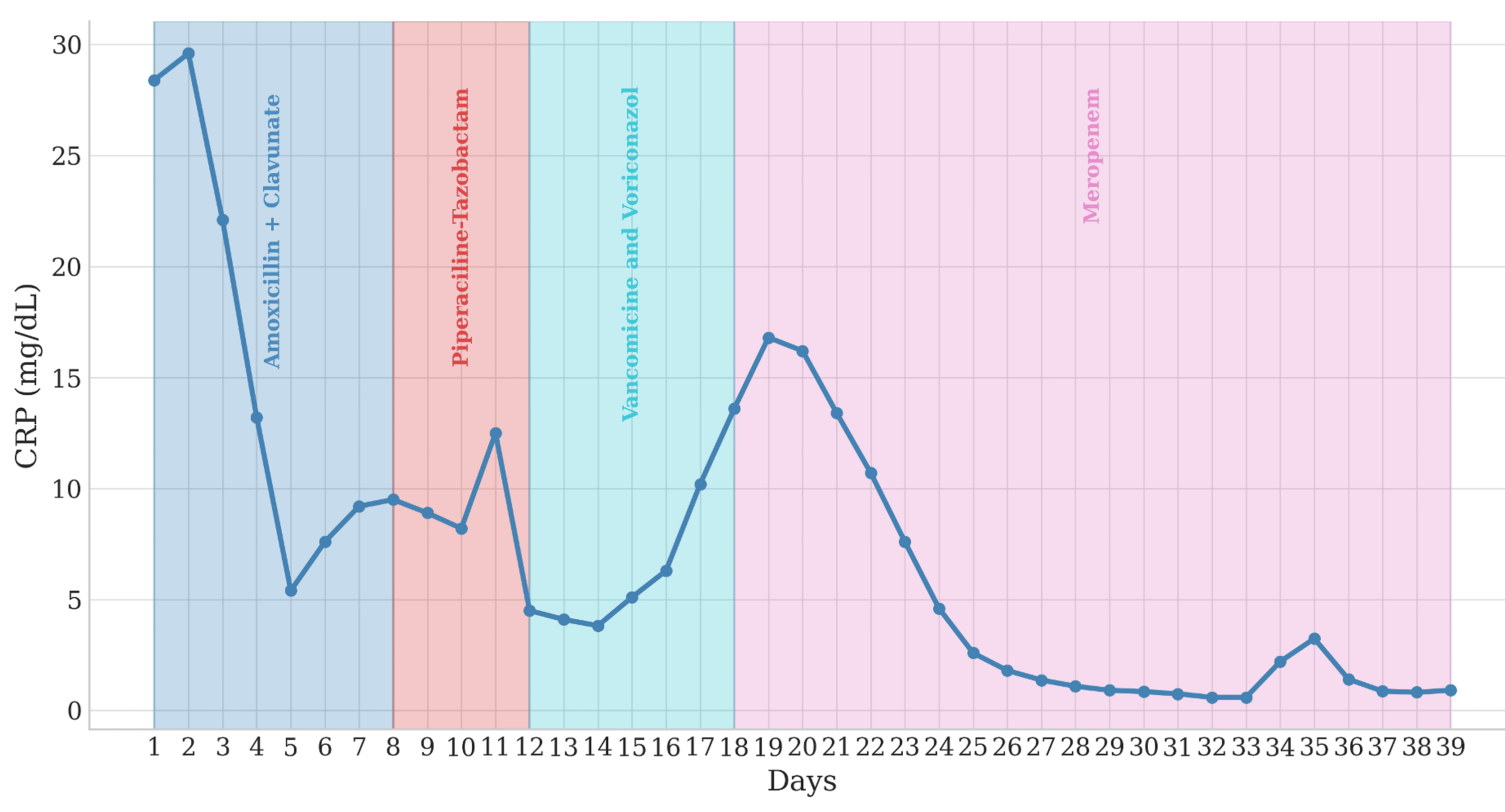
Temporal evolution of C-reactive protein (CRP) levels showing correlation with antibiotic therapy changes

During the NIVU stay, recurrent fever and hemodynamic instability developed, necessitating norepinephrine (maximum 0.05 mcg/kg/min) and high-flow nasal cannula oxygen (60 L/min, fraction of inspired oxygen (FiO₂) 60%). Empyema was excluded by thoracentesis, and empiric meropenem was initiated. Repeat sputum and blood cultures were obtained during each episode of clinical deterioration, all of which remained negative. Pleural fluid cultures were not performed. Bronchoscopy with bronchoalveolar lavage (BAL)excluded MRSA and fungal infection (negative galactomannan antigen). Vancomycin and voriconazole were discontinued after 6 days due to lack of response. Following initiation of meropenem, marked clinical improvement was observed, including resolution of fever, declining inflammatory markers, and reduced oxygen requirements (Figure 6).

**Figure 7:**
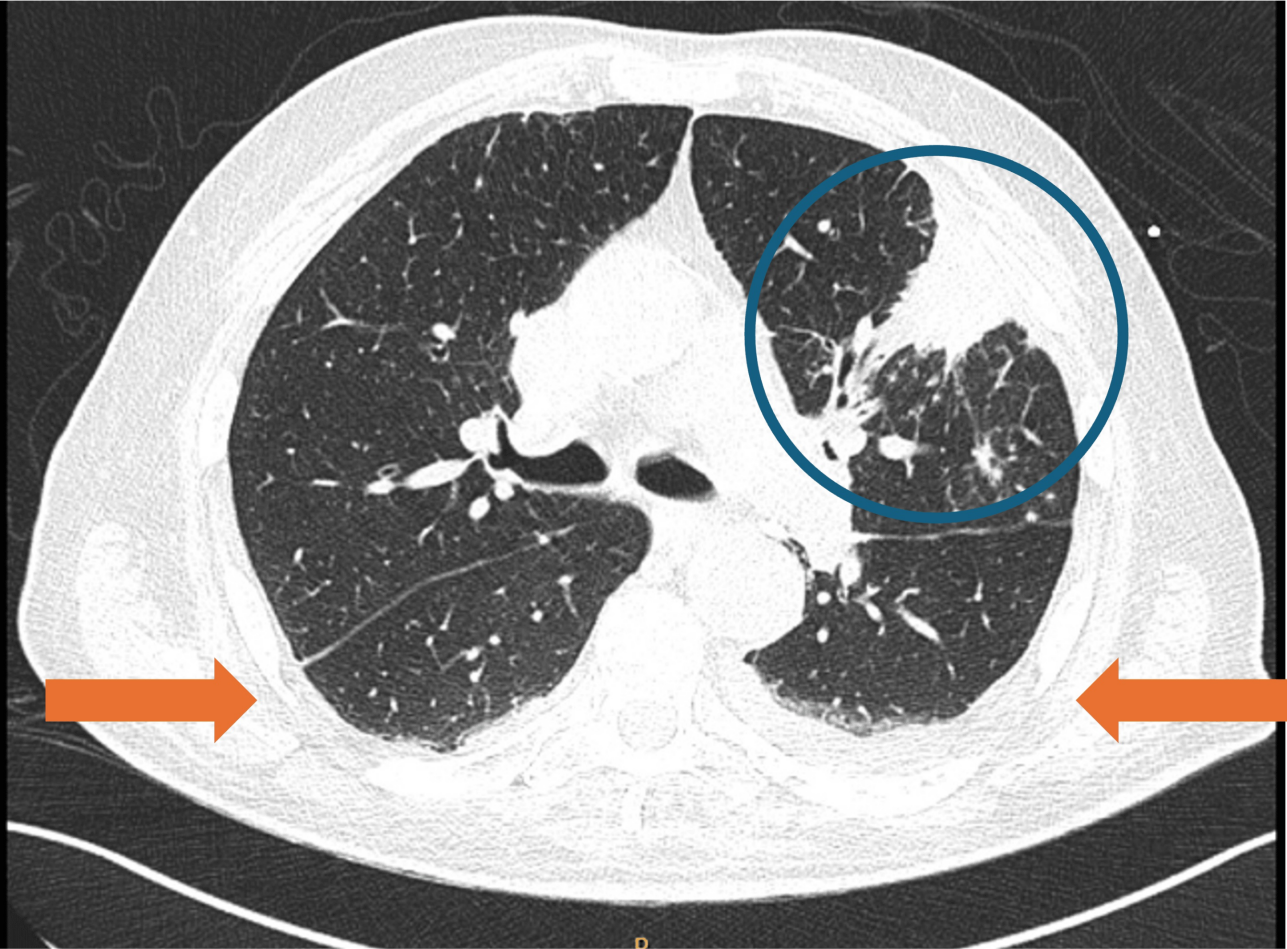
Axial chest CT demonstrating improvement of left upper-lobe consolidation with reduction of the cavitary component (blue circle) and decreased bilateral pleural effusions (orange arrows).

**Figure 8:**
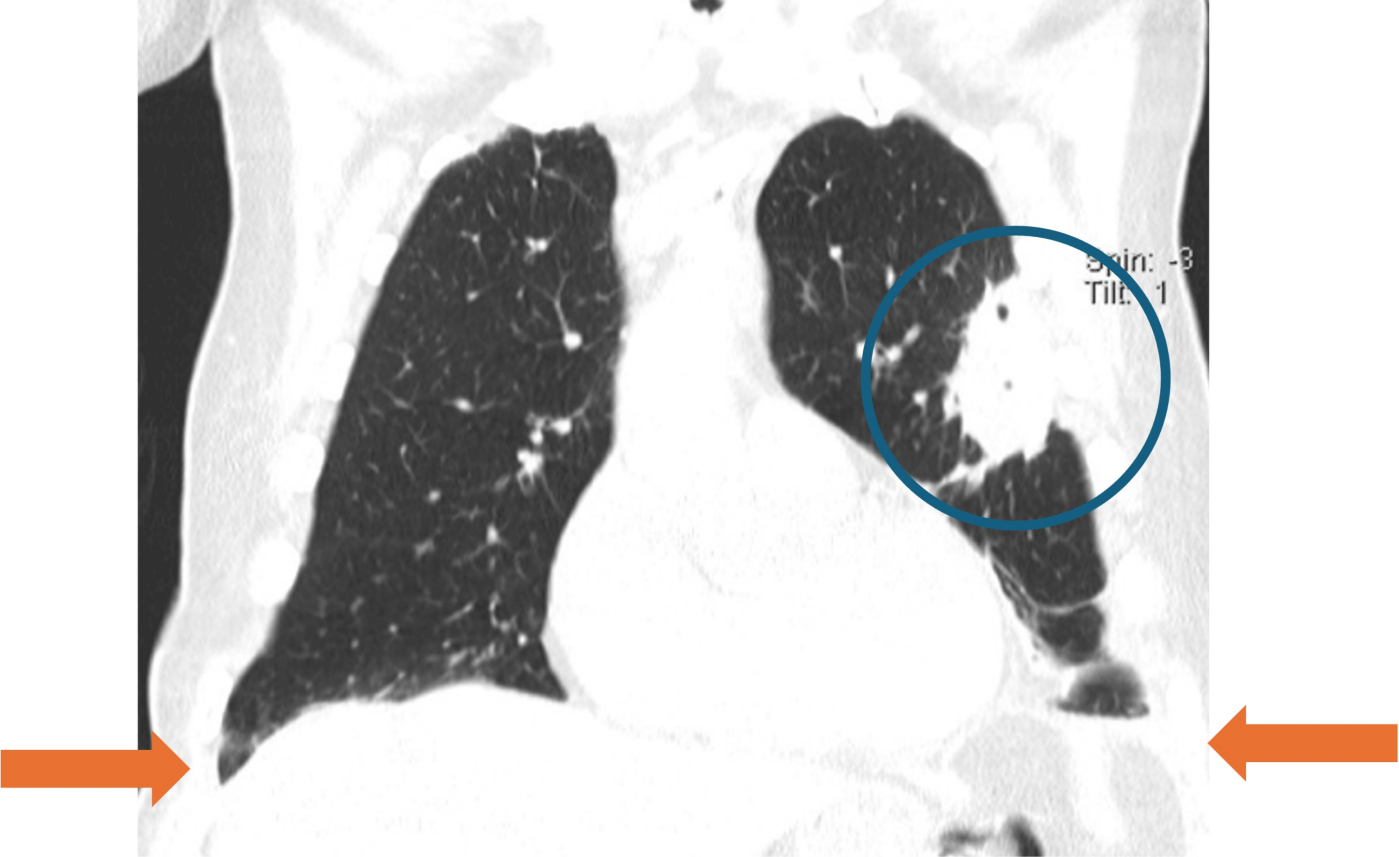
Coronal chest CT demonstrating interval improvement of the left upper-lobe consolidation with reduction of the cavitary component (blue circle), along with decreased bilateral pleural effusions (orange arrows).

After 11 days of vasopressor support, hemodynamic stability was achieved, and the patient was transferred to the medical ward on day 32 of illness. Oxygen therapy was discontinued on day 37, and the patient remained eupneic on room air. A 21-day course of meropenem was completed. Follow-up CT demonstrated regression of left upper-lobe cavitation, resolution of right lower-lobe infiltrates, and reduced pleural effusions (Figures 7, 8).

Due to the atypical severity and progression of the respiratory infection, comprehensive evaluations were conducted to identify potential underlying causes of immunosuppression predisposing to necrotizing pneumonia. During hospitalization, targeted screening for secondary immunodeficiency was performed, including assessments for autoimmune diseases, hematologic malignancies (Table 2), and chronic infections (Table 3), which were negative. Glycated hemoglobin was 5.9%, excluding diabetes mellitus, and transthoracic echocardiography ruled out structural heart disease.

**Table 2:** Autoimmune and hematologic screening results during hospitalization Abbreviations: Anti-Ro, Sjögren's syndrome antigen A; Anti-La, Sjögren's syndrome antigen B; Anti-SM, Smith antigen; Anti-U1-RNP, ribonucleoprotein; Anti-Scl-70, topoisomerase I; Anti-Jo-1, histidyl–tRNA synthetase; LKM-1, liver kidney microsomal type 1 antibodies; LC-1, liver cytosol type 1 antibodies; SLA/LP, soluble liver antigen/liver pancreas antibodies; AMA-M2, anti-mitochondrial antibody M2; AMA-M2-3E BPO, fusion antigen; Sp100, Sp100 nuclear antigen; PML, promyelocytic leukemia protein; gp210, glycoprotein 210, dsDNA, double-stranded DNA.

Autoimmune and hematologic panel	Normal range	Result
Complement C3 (mg/dL)	90-180	118
Complement C4 (mg/ dL)	10-40	23.3
Rheumatoid factor (IU/L)	<15	<15
Anti-cyclic citrullinated peptide antibodies (u/ml)	<5	<5
Anti-nuclear antibodies	Negative	Negative
Anti-dsDNA antibodies	Negative	Negative
Anti-Ro/La/Sm/U1-RNP/Scl-70/Jo-1	Negative	Negative
Anti-hepatic antigens antibodies (LKM-1, LC-1, SLA/LP, AMA-M2, AMA-M2-3E, Sp 100, PML, gp210)	Negative	Negative
Anti-neutrophil cytoplasmic antibody (c-ANCA, p-ANCA)	Negative	Negative
Angiotensin-converting enzyme	Negative	Negative
Serum protein electrophoresis	Inflammatory pattern and no monoclonal spike	
Beta-2 microglobulin (mg/dL)	1.09-2.53	1.51
Kappa free light chains (mg/L)	3.3–19.4	24.5
Lambda free light chains (mg/L)	5.7- 26.3	39,0
Kappa/Lambda ratio	0.26-0.65	0,628
IgG (mg/dL)	600−1600	1160,0
IgA (mg/dL)	80−450	418,0
IgM (mg/L)	50-200	24,5

**Table 3:** Serological screening for chronic viral and mycobacterial infections performed during hospitalization.

Infectious agent	Result
Hepatitis B surface antigen	Negative
Hepatitis B surface antibody HBs	Negative
Hepatitis B core antibody	Negative
Hepatitis C antibody	Negative
Antibodies against HIV-1 and HIV-2	Negative
Interferon gamma release assay (IGRA)	Negative

The patient was discharged in stable condition after a 39-day hospital stay and remained asymptomatic at pulmonary follow-up in February 2023. Imaging revealed mild residual fibrosis in the left upper lobe.

## Discussion

Necrotizing pneumonia (NP) is a severe condition that necessitates immediate and aggressive intervention because of its high mortality risk. The estimated prevalence of NP is 10-12%. NP is defined by progressive parenchymal necrosis, liquefaction, and cavitary destruction within consolidated lung tissue [1]. 

The underlying pathogenesis involves severe inflammatory injury, resulting in vascular thrombosis, tissue ischemia, and impaired antibiotic penetration [3]. 

Elderly patients and individuals with chronic lung conditions like COPD are especially at risk because of weakened airway defenses. This increases their likelihood of experiencing severe, sometimes polymicrobial infections, delayed recovery, and complications such as necrotizing pneumonia [4]. 

Necrotizing pneumonia may initially resemble severe community-acquired pneumonia, with necrotizing changes becoming apparent as the disease progresses [5]. CT scans are the best way to confirm NP, as they show the lung damage and cavities [6]. In our patient, repeated CT scans helped show that the disease was getting worse, even though he seemed better at first. 

Laboratory tests did not identify the specific bacteria causing the infection, which is common in NP and makes it difficult to determine whether the problem is a recurrence of the initial infection or a new hospital-acquired infection [6]. 

There are currently no standardized protocols for NP management, which relies on clinical judgment, multidisciplinary input, and antimicrobial stewardship. Empiric broad-spectrum antibiotics should be individualized and frequently reassessed based on patient evolution and, when available, microbiological data [7]. 

The patient initially received amoxicillin-clavulanate for MSSA, with only transient improvement. Clinical deterioration necessitated escalation to broader-spectrum agents, including piperacillin-tazobactam, vancomycin, and voriconazole, but without clinical benefit. Although clinical improvement was noted after initiation of meropenem, attributing this response solely to infection with multidrug-resistant Gram-negative organisms remains speculative in the absence of microbiological confirmation or local resistance data. Alternative explanations, such as delayed antibiotic response, improved lung penetration, or cumulative antimicrobial effects, cannot be excluded. The decision to escalate to carbapenem therapy should be carefully considered and always guided by antimicrobial stewardship principles to avoid the risks associated with unnecessary early use of broad-spectrum agents, especially when multidrug resistance is not confirmed [8]. 

Necrotizing pneumonia can be very dangerous, with death rates as high as 45% in severe cases, especially for patients with other health issues. However, careful monitoring and being ready to change treatment can lead to recovery, even for high-risk patients [7]. Despite being older and having COPD, our patient recovered fully with medication alone. His case shows that promptly recognizing changes on scans and adjusting antibiotics can prevent serious problems. 

Necrotizing pneumonia is a life-threatening complication of bacterial pneumonia that requires early diagnosis, close monitoring, and aggressive antimicrobial management. Clinical deterioration despite appropriate therapy should prompt suspicion of superinfection with resistant organisms. Empiric escalation to carbapenem coverage, guided by imaging findings, clinical response, and local antimicrobial resistance patterns, may be lifesaving in selected patients.

## Conclusions

Necrotizing pneumonia remains a severe complication of bacterial pneumonia, with significant morbidity and mortality, particularly in elderly patients with chronic lung disease. This case highlights the importance of dynamic clinical reassessment and timely recognition of radiologic progression. While escalation of antimicrobial therapy may be warranted in the setting of treatment failure, recommendations regarding empiric carbapenem use should be made cautiously. In the absence of microbiological confirmation, the observed clinical improvement with meropenem in this case should be interpreted as hypothesis-generating rather than practice-changing.
